# Mechanisms and Drug Therapies of Bioprosthetic Heart Valve Calcification

**DOI:** 10.3389/fphar.2022.909801

**Published:** 2022-06-03

**Authors:** Shuyu Wen, Ying Zhou, Wai Yen Yim, Shijie Wang, Li Xu, Jiawei Shi, Weihua Qiao, Nianguo Dong

**Affiliations:** Department of Cardiovascular Surgery, Union Hospital, Tongji Medical College, Huazhong University of Science and Technology, Wuhan, China

**Keywords:** bioprosthetic heart valve, ectopic calcification, structural valve degeneration, mechanisms, drug therapy

## Abstract

Valve replacement is the main therapy for valvular heart disease, in which a diseased valve is replaced by mechanical heart valve (MHV) or bioprosthetic heart valve (BHV). Since the 2000s, BHV surpassed MHV as the leading option of prosthetic valve substitute because of its excellent hemocompatible and hemodynamic properties. However, BHV is apt to structural valve degeneration (SVD), resulting in limited durability. Calcification is the most frequent presentation and the core pathophysiological process of SVD. Understanding the basic mechanisms of BHV calcification is an essential prerequisite to address the limited-durability issues. In this narrative review, we provide a comprehensive summary about the mechanisms of BHV calcification on 1) composition and site of calcifications; 2) material-associated mechanisms; 3) host-associated mechanisms, including immune response and foreign body reaction, oxidative stress, metabolic disorder, and thrombosis. Strategies that target these mechanisms may be explored for novel drug therapy to prevent or delay BHV calcification.

## 1 Introduction

Valvular heart disease (VHD) inflicts a heavy burden on global health care, with an incidence rate of 13.3% for people over 75 (Roger et al., 2011; Virani et al., 2021). Given the aging of population worldwide, the prevalence of VHD is expected to rise exponentially and will double before 2050 (D’arcy et al., 2016; Dziadzko et al., 2018). Currently, surgery replacement of the dysfunctional native valves with artificial valves is the standard therapy for VHD, and artificial valves generally fall into one of the two categories: mechanical heart valve (MHV) or bioprosthetic heart valve (BHV) (Vahanian et al., 2021). The annual demand for interventions is expected to hit 850,000 by 2050, owing to the increasing prevalence of VHD (Yacoub and Takkenberg, 2005). In the past 2 decades, the application of BHV has surpassed MHV. Rapid advances in the field of transcatheter aortic valve replacement (TAVR) also contributed to the extending scope and appreciation for BHV (Wen et al., 2020).

In contrast to MHV, BHV has significant advantages by eliminating the need for anticoagulation therapy while possessing exquisite hemodynamic properties similar to those of native valves. However, its durability was hampered by inevitable structural valve degeneration (SVD). In brief, SVD is defined as a permanent intrinsic change of the valve resulting in calcification, leaflet tear, pannus deposition of a valve, which eventually manifested as stenosis or regurgitation prompting high-risk reintervention (Capodanno et al., 2017; Dvir et al., 2018). Thus, SVD is becoming a major issue for surgeons and researchers. Calcification is the most prevalent pathological form of SVD (Koziarz et al., 2020) and is believed to be the final pathway for valve dysfunction, leading to progressive cusp stiffness and obstruction as well as leaflet fragility (Cartlidge et al., 2019). Therefore, we review current knowledge of the pathogenesis for BHV calcification.

BHV calcification has been once considered a passive degenerative process, but now is seen as a complex mechanism actively regulated by several factors (Kostyunin A. E. et al., 2020). Recent studies provided evidence that multiple processes were involved in BHV calcification, including glutaraldehyde (GLUT) pretreatment, material composition, mechanical stress, and immune response. In this review, we sought to clarify pathophysiological features and mechanisms of BHV calcification as well as potential drug strategies to prevent or delay BHV calcification.

## 2 Types of BHV

Since the 1960s, techniques and technologies of surgical aortic valve replacement as well as the implanted aortic valves themselves have been flourishing. Despite the superior long-term durability, patients fitted with MHV face the burden of lifelong anticoagulant treatment.

In the recent 2 decades, with the advent of TAVR and the improvement of BHV, there has been a substantial shift toward the use of BHVs compared to MHVs. Studies have demonstrated that the overall usage of BHV in isolated aortic valve procedures was up to 87% (Beckmann et al., 2016). Generally, BHV can be classified according to materials that derived from pulmonary autografts, homografts, and xenografts. Xenografts are the main materials of commercial BHV, so the discussion we present below focuses on xenografts BHV. Conventionally, porcine aortic valve leaflets or pericardial bovine patches used for BHV are preserved by GLUT fixation and other anti-mineralization treatments, partly preventing immunogenicity and improving durability. Of note, prior studies have shown that pericardial bovine valves have significantly better hemodynamic results with lower gradient pressure and larger orifice areas than porcine valves (Ruzicka et al., 2009; Yap et al., 2013). In order to serve the needs of various innovative technologies and pathoanatomical diversity of the aortic roots, the design of BHV can be subdivided into stented or stentless surgical valve and balloon-expandable or self-expanding transcatheter valve. Stented valve is composed of polymeric material or scallop-shaped external sewing ring located outside of the stent frame, while stentless valve have neither a stent frame nor a base ring that supports valve leaflets providing greater effective orifice areas and lower transprosthetic gradients (Paradis et al., 2015; Dangas et al., 2016).

## 3 Durability and Failure of BHV

Despite the many advantages of BHV over MHV, particularly without the need for lifelong anticoagulative treatment, BHV is still not devoid of shortcoming. Numerous cohort studies indicate the existing commercial BHV are not fully addressing long-term needs as a prosthetic valve substitute due to inevitable SVD. A retrospective study (Forcillo et al., 2013) reported the freedom rate from reoperation after implanted Carpentier-Edwards valve due to prosthesis dysfunction averaged 98 % ± 0.2%, 96% ± 1%, and 67% ± 4% at 5, 10, and 20 years, respectively. Tirone et al. (David et al., 2010) evaluated 1,134 patients underwent aortic valve replacement surgery with Hancock II bioprosthesis, showing survival rate and freedom rate from SVD at 20 years were 19.2% ± 2% and 63.4% ± 4.2%, respectively. The mean duration of SVD after implantation of Mitroflow bioprosthesis was only 3.8 ± 1.4 years (Senage et al., 2014). Therefore, Long-term outcomes of surgical BHV remain suboptimal, irrespective of their brands and special anti-calcification pretreatment.

Since TAVR has only been widely generalized after 2007, studies for durability of transcatheter valves are almost circumscribed to the first 5 years of follow-up. Five-year rate of BHV dysfunction undergoing TAVR was 1.4% (Barbanti et al., 2015). Noteworthy, SVD usually begins 8 years after implantation, with an increasing rate of SVD after 10 years (Dvir et al., 2018). Generally, transcatheter valves are assumed to have even worse durability compared with surgical valves due to several factors. Transcatheter valves are thinner than surgical valve to permit transcatheter delivery. Moreover, transcatheter valves were under higher mechanical stresses and strains because of non-circular, asymmetric stent deployment. Transcatheter valves is vulnerable to traumatic injury during implantation, while surgical valves remain well conserved without any contact during operation. In this context, the SVD rate of transcatheter valves was substantially underestimated.

Limited durability severely impedes broadening the scope of BHV usage. Great care should be taken to decide whether to choose BHV before surgery, especially for patients with extremely high risks for SVD. Multiple factors are associated with early SVD onset, including young age of patients, end-stage renal disease, diabetes mellitus, hyperparathyroidism, smoking, and prosthesis-patient mismatch (Kostyunin A. E. et al., 2020; Ochi et al., 2020), which suggest that BHV failure is a continuous variable process, rather than a binary categorical parameter. Given the similarities of risk factors of BHV failure, atherosclerosis, and calcific aortic valve disease, they may share the same molecular mechanisms, in which calcification is the core signature and important target for intervention. Understanding the biomolecular mechanisms related to BHV calcification is the essential first step to explore potential therapeutic targets to inhibit or at least slow the progression of SVD and open novel avenues for improving the longevity of BHV to fulfill clinical requirements.

## 4 General Features of BHV Calcification

**FIGURE 1:**
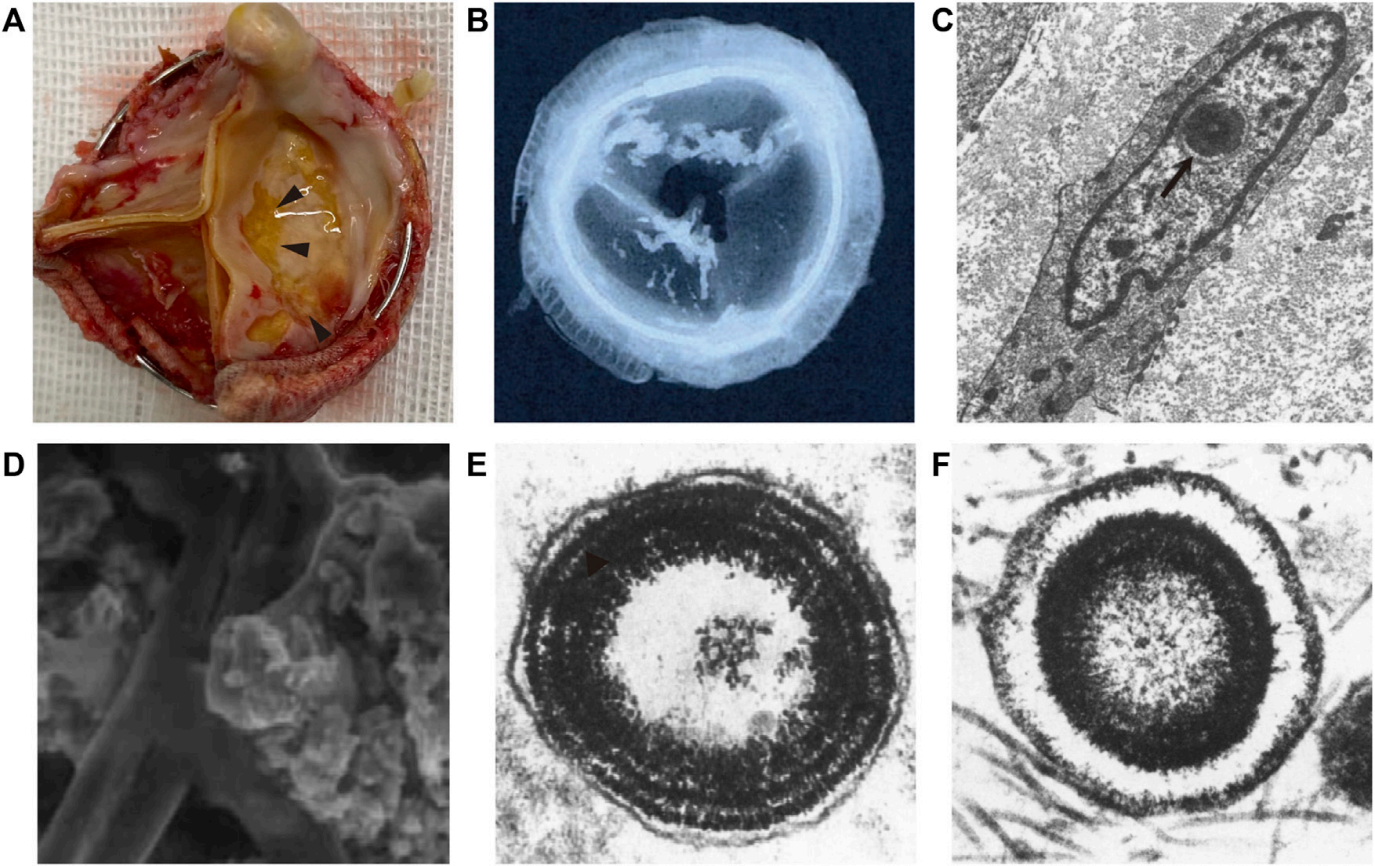
Schematic of bioprosthetic heart valve calcification. **(A)** Gross view of bioprosthetic heart valve calcification (arrowhead). **(B)** Low-energy radiography of bioprosthetic heart valve calcification (Delogne et al., 2007). **(C)** Ultrastructure of calcium deposits in the cell nuclei (arrow) (Schoen et al., 1994). **(D)** Scanned electron microscopy view of calcific loci depositing on collagen and elastin (Delogne et al., 2007). **(E,F)** calcospherulae arranged in concentric rings with and without a central core (Valente et al., 1985).

BHV calcification is one form of ectopic calcification, referring to the aberrant deposition of calcium phosphate complexes in soft tissues (Figures 1A,B). Based on the pathogenic mechanisms, ectopic calcification can be classified as dystrophic, metastatic, idiopathic, iatrogenic, or tumoral (Chander and Gordon, 2012; Li et al., 2014; Boraldi et al., 2021). In fact, to date, the pathogenesis of BHV calcification remains unclear. However, the determinants of all kinds of ectopic calcification cannot be separated from the original level of calcium, the presence of scaffolding for mineral deposition, and abnormal regulations during calcification. This chapter will draw a comprehensive picture of BHV calcification as possible from 1) the composition of the calcific foci in BHV; 2) micro and macro-level perceptions for BHV calcification sites. Compositions and sites of calcific foci are not only the final manifestation but could reflect mechanisms of BHV calcification.

### 4.1 The Composition of Calcifications

Calcium phosphate (CaP) is the common name of the calcific deposits family, and different type of CaP is formed under different physiological and pathophysiological situations (Eliaz and Metoki, 2017). Although several studies have confirmed the mineral salt of calcific BHV is a mixture of CaP, the major components of BHV calcifications from different studies were inconsistent and conflicting. Delogne et al. (2007). showed spectral features very similar to a crystalline hydroxyapatite (HAP) spectrum, and refuted the findings of Mikroulis et al. (2002), who reported the presence of dicalcium phosphate dihydrate (DCPD), octacalcium phosphate (OCP), and *β*-tricalcium phosphate (*β*-TCP). However, Tomazic et al. (1994) characterized calcific deposits from 10 failed BHV that had been implanted in patients for 2–13 years and suggested BHV calcifications were composed of either an apatitic and/or OCP-like material, but also eliminated HAP as a significant fraction in BHV calcifications due to measured refraction index. CaP mixture complexity and significant individual differences contribute to different results. Despite these differences, all of these studies collectively highlight that the Ca/P molar ratio of BHV calcific deposits ranges from 1.34 to 1.67, considerably lower than 1.70 found in mature atherosclerotic plaque, natural valve calcification, and mature skeletal (Tomazic et al., 1994; Mikroulis et al., 2002; Delogne et al., 2007). Collectively, it is worth raising the possibility of the presence of precursor phases associated during the early stages of calcification with substantial incorporation of sodium, magnesium, silicon, and carbonate. Regardless, different condition contributes to a different type of CaP deposited. Thus, further studies in the component of BHV calcification may help make the BHV calcification pathology progression clearer.

### 4.2 The Location of CaP

Some ultrastructures of cell and extracellular matrix (ECM) provide scaffolding or abundant feedstock for calcium deposits that are prone to calcification. Despite differences in the infrastructure of the porcine aortic valve and bovine pericardium, the site features of calcification of these materials were virtually similar (Schoen et al., 1986). Initial calcification deposits were localized predominantly to cell remnants (Valente et al., 1985; Schoen et al., 1986; Schoen et al., 1994). Microscopically, the earliest deposits were noted in the nuclei, but it also appears to be at residual organelles or associated with the plasma membrane in the cytoplasm (Schoen et al., 1994). In addition, calcific deposits are also involved in the fibrosa, and later deposits expanded the spongiosa. Namely, cellular debris, collagen, and elastin can serve as foci for calcification (Figures 1C,D). The concrete mechanisms for this progression are described below. When considering BHV as a whole, cuspal commissures and basal attachment sites are more susceptible to calcification (Schoen et al., 1994; Schoen and Levy, 1999). Different implanted positions could be expected to have different degrees of BHV calcification, as calcification developed more commonly in inflow valves (ventricular valve) than the outflow valve (aortic valve) (Liao et al., 2008), which may be ascribed to different mechanical stress.

## 5 Pathophysiological Mechanism of BHV Calcification

**FIGURE 2:**
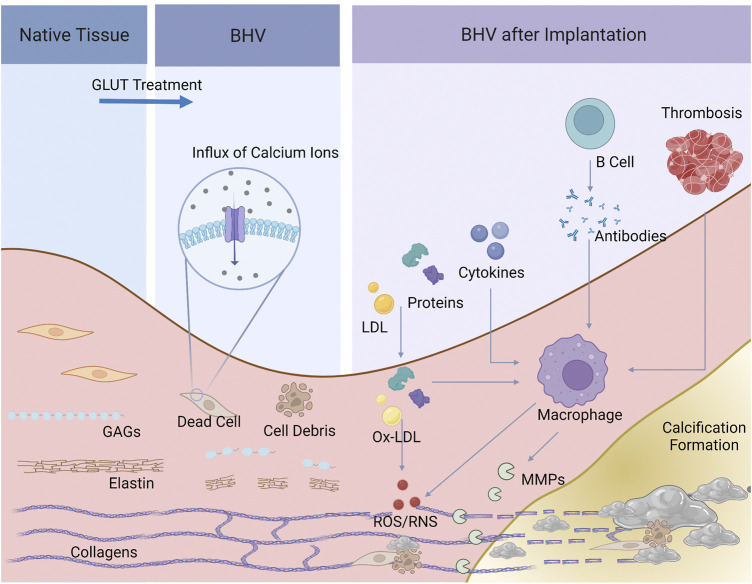
Schematic of the process of BHV calcification. Dead cells and cell debris, and elastin and GAGs degradation, and collagen crosslinks were present after GLUT treatment, providing calcium ions and the specific space structure for calcification. Serum protein and lipid infiltrated, cytokines, xenoantibodies secreted by B cells and thrombosis would activate macrophages and induce inflammatory response. Macrophages further secret MMPs and product ROS/RNS, leading to BHV calcification.

By analogy with the pathology of native valve calcification, the mechanism of BHV calcification should not be entirely attributed to a passive process of calcium deposits but is probably complex and multifactorial, and a comprehensive understanding remains elusive. In this review, we categorized BHV calcification mechanism into two major categories: material-associated mechanism and host-associated mechanism. The former involves the specific nature of valve materials and physicochemical properties that lead to the high susceptibility of calcification. On the other hand, the host-associated mechanism is implicated in several processes after BHV implantation, such as protein adsorption, oxidative stress, activation of immune systems, and local inflammatory response (Figure 2).

### 5.1 Material-Associated Mechanism

#### 5.1.1 GLUT and Cell Debris

GLUT efficiently crosslinks collagen and mask xenoantigens, showing substantial advantages like no other crosslinkers. However, the mechanism of BHV calcification induced by GLUT has been corroborated (Kim et al., 1999; Schoen and Levy, 1999). Among these, calcification of implanted BHV is mainly due to cytotoxic effects treated by GLUT. Under normal physiological circumstances, the intracellular calcium level is up to a thousand-fold lower than the extracellular one (Kiełbik et al., 2020), tightly maintained by the mitochondria through the calcium pump. *In-vitro* studies revealed that GLUT treatment causes cell death and inactivation of calcium pump, triggering an immediate influx of calcium ions from extracellular spaces and a rise of intracellular phosphate ions in a dose-dependent manner (Kim et al., 1999). However, due to very poor vascularization in implanted BHV tissue, cell debris cannot be promptly scavenged by macrophages. In this scenario, cell-membrane-derived acidic phospholipids form the so-called “calcium-phospholipid-phosphate complexes” (Boskey and Posner, 1976; Schoen and Levy, 1992), so that calcium appears to deposit in the cell debris. Another major mineral nucleation site is involved in cell-derived matrix vesicles, the production of cell byproducts, including multiply undefined forms, like “matrix vesicles”, “apoptotic bodies”, “exosomes” et al. (Schraer and Gay, 1977). These initial calcific foci consist of concentrically arranged, multi-laminated vesicular bodies that were named spherulites or calcospherulae (Figures 1E,F) (Valente et al., 1985; Lee, 1993), which have been reported to have robust facilitatory effects on mineral deposition. Moreover, free aldehyde groups and impairing charges balances after GLUT treatment may hence promote calcification as well, and aldehyde-free treatment was an effective method to enhance the anti-calcification properties (Chen et al., 1994; Rodriguez-Gabella et al., 2017; Meuris et al., 2018; Badria et al., 2020).

#### 5.1.2 Extracellular Matrix Damage

ECM, mainly composed of fibrillar collagens, elastin, and glycosaminoglycans (GAGs), is the largest source of free calcium ions during the process of BHV calcification. Of concern, mineralization of ECM is a secondary process that occurs after collagen and elastin fibers were embedded by dead cells or vesicles induced calcium deposition (Kim, 2002). GLUT-fixed tissues are not entirely resistant to enzymatic attack and are not metabolically inert. Degradation and breakdown of ECM provide spatial facilitation for calcification. GAGs provide hydration and lubrication in the native valves and are important for the stable assembly of the ECM. In particular, GAGs interact specifically with type I collagen fibrils, bridging and stabilizing adjacent collagen fibrils (MacGrogan et al., 2014). Nevertheless, GAGs cannot be crosslinked by GLUT, and are gradually lost during the preparation, storage, and implantation of BHV (Leong et al., 2013). Degradation of GAGs disrupts collagen integrity, resulting in exposing hole zones, a 3-dimensional structure favoring nucleation of CaP crystals. In parallel, GLUT doesn’t react with elastin. And when undergoing proteolysis, elastin possesses a special structure that smooths the path of calcium deposition (Bailey et al., 2003; Simionescu et al., 2003; Wang et al., 2015). Multiple studies suggested that stabilizing GAGs and elastin help reduce BHV calcification (Ohri et al., 2004; Raghavan et al., 2007; Leong et al., 2013; Lei et al., 2019). Except for degradation, fibers ruptured and damaged by mechanical stress are also presented with calcium-binding sites (Whelan et al., 2021).

In short, the residual cell debris and cell-derived matrix vesicles are the primary loci of calcification in BHV, while the ECM provides massive calcium ions and a specific space structure for mineralization during the degradation.

### 5.2 Host-Associated Mechanism

#### 5.2.1 Immune/Inflammatory Response

Young patient age has been considered as an exact risk factor for early SVD of BHV (Dvir et al., 2018; Pibarot et al., 2020). The predicted 15-year risk of needing reoperation because of SVD is 50% for patients at age of 20, but patients >65 years old may show greater freedom from SVD (Chan et al., 2006; Siddiqui et al., 2009; Otto et al., 2021a). This phenomenon is widely believed to be attributable to immune response because young adults mount a more vigorous immune response than elderly people. In fact, mounting evidence has accumulated over the past decade that strongly points toward a crucial involvement of immune response in the calcification and degradation of BHV (Hopkins, 2006; Siddiqui et al., 2009; Manji et al., 2015b; Costa, 2020). Significantly increased immune cellular infiltration, including T cells, macrophages, B cells, neutrophils, and plasma cells, was observed in the implanted calcific BHV tissue, with an elevated cytokine concentration accompanied (Manji et al., 2015a; Bozso et al., 2021). Senage and his colleagues (Senage et al., 2022) carried out a large cohort study demonstrating graft-special antibodies significantly increase and deposit on calcific BHV tissue 1 month after BHV implantation. Animal studies also showed T cells and macrophages infiltration and antibody rise in GLUT fixed valve tissues (Manji et al., 2006). The above studies indicate that inflammatory reaction and immune response may play vital roles in BHV calcification processes.

BHV implantation necessarily induces foreign body reactions. Foreign body reaction is an immune-mediated reaction to implanted materials where a cascade of inflammatory events results in granuloma and fibrous encapsulation (Veiseh and Vegas, 2019). Different from other biomaterials, the implanted BHV does not form a visible fibrous capsule under high shear forces and complex haemodynamic profile, but allows non-specific protein adsorption (Shen et al., 2001; Sakaue et al., 2018) and inflammatory cells infiltrating (Kostyunin A. et al., 2020). Host plasma proteins adsorption leads to a series of subsequent effects including complement system and platelet activation, coagulation cascade, and cell adherence (Zhu et al., 2019). Moreover, Antonio et al. (Frasca et al., 2020) have found that human serum albumin and glycation infiltration lead to BHV tissue matrix disruption and change the biomechanical properties of valve leaflets. In detail, glycation end products not only alter collagen fiber interactions, potentially causing leaflet stiffening, but result in modulation of cell phenotypes and instigation of inflammation via glycation product-mediated receptor signaling (Frasca et al., 2020).

Subsequently, monocyte/macrophages are recruited by the layer of protein adsorbed onto the valve surface. Through the electron microscopy visualization of calcified BHV, Alexander et al. (Kostyunin A. et al., 2020) have elaborately illustrated a multi-step process that monocyte infiltration followed by a macrophage-driven ECM disintegration. Monocytes/macrophages roll and then adhere to the surface of BHV, tearing the ECM proteins by invadopodia. Moreover, (neo)vascularization facilitates the migration of macrophages and other immune cells and alters microenvironmental pH as well as bring available mineral ions (Kitagawa et al., 2015; Kostyunin A. et al., 2020; Katsi et al., 2021). Infiltrated macrophages concentrate the cytoplasmic granules at the leading edge of the cells and release proteolytic enzymes, such as matrix metalloproteinases (MMP) (Simionescu et al., 1996; Simionescu et al., 2003), and plasminogen (Sakaue et al., 2018), leading to degradation and delamination of the ECM (Kostyunin A. et al., 2020). MMP-2 and MMP-9 were markedly elevated in the calcified BHV tissues, compared to the noncalcified BHV. MMPs could degrade partially GLUT-fixed collagen and particularly large amounts of elastin. Furthermore, MMPs play a vital role in the promotion of inflammatory, fibrotic, and osteogenic genes overexpression (Matilla et al., 2020). In addition, plasminogen was strongly stained in CD68-positive macrophages among calcified BHV (Sakaue et al., 2018). To our knowledge, plasminogen, acting as a potent proinflammatory mediator, contributes to the induction of cytokines and intracellular signaling events and stimulates the activation of macrophages (Shen et al., 2012). Under these circumstances, the strong MMP-dependent proteolysis and the fibrinolytic system can cleave most ECM proteins (Kostyunin A. E. et al., 2020).

Besides, macrophages also secret calcium-binding proteins, such as osteopontin and osteonectin, which are adapted for engendering BHV calcification. Osteonectin, also known as BM-40 or SPARC (secreted protein acidic and rich in cysteine) (Wang et al., 2006), has a considerable high affinity to calcium (Busch et al., 2000). Osteonectin also modulates cell function by interacting with cell-surface receptors, metalloproteinases, growth factors, and other bioeffector molecules, involved in tissue remodeling, repair, development, and cell turnover (Bradshaw and Sage, 2001; Zhu et al., 2020). Given to macrophage-derived matrix vesicles contribute to microcalcification in atherosclerotic plaques (New et al., 2013), macrophages may also secrete extracellular vesicles capable of inducing BHV mineralization.

Xenoantigens are the primary cause of provoking adaptive immune reactions after BHV implantation. Although GLUT fixation and other pretreatments minimize the immunological determinants of bioprosthetic leaflet tissue to avoid xenograft rejection, the hurdle still exists because the immunogenicity of such tissue is not sufficiently abolished, especially carbohydrate antigens. It is well-established that the alpha-gal epitope is the dominant mediator in discordant xenoimplants (Naso et al., 2013; Kim et al., 2015; Li, 2019). In this regard, Gal-knockout BHV represented a novel and potentially useful strategy for reducing BHV calcification (Lila et al., 2010; Park et al., 2010; McGregor et al., 2013). The additional two immunogenic carbohydrate antigens that have been identified are N-glycolylneuramic acid (Neu5Gc) antigen (Lee et al., 2016; Reuven et al., 2016) and the Sid blood group antigen (Sda) (Li, 2019). Apart from carbohydrate antigens, Katherine et al. (Gates et al., 2019) have identified 19 specific protein antigens from GLUT fixed bovine pericardial heart valves that stimulate the graft-specific humoral immune response in patients. Intriguingly, they found calcium-binding proteins were the most highly over-represented biological function of antigens, but such antibody-binding effect of those proteins on BHV calcification is yet to be elucidated.

In the humoral response triggered by unmasked xenoantigens, pre-existing antibodies play a vital role in the opsonization of inflammatory cells to recruitment and proliferation, phagocytosis, efferocytosis, etc, thereby distinctly facilitating the overall immune response. Inflammatory cells, such as neutrophils and macrophages, adhesion onto BHV tissue surface and subsequently infiltrate into the leaflets, releasing stored MMPs (Ground et al., 2021). Similarly, cellular immunity participates in BHV calcification. Histological studies of BHV removed from patients showed leukocytes destroying collagen fibers, with crystalline material present on their surfaces, suggesting it may have been acting as a nidus for calcification (Stein et al., 1988). Regardless, humoral or cellular immunity would decidedly undermine the integrity of valve, leading to exposure to calcification site or increasement in calcification composition.

#### 5.2.2 Oxidative Stress

It is well known that reactive oxygen and nitrogen species (ROS/RNS) have a potentially severe impact on both host and implanted biomaterials. ROS/RNS are continuously generated as normal by-products of cell metabolism and act as signaling molecules at lower concentrations controlling cell proliferation and differentiation in many cell types (Manolagas, 2010). But the excessive productions of ROS/RNS participate in numerous pathogenesis of diseases including cancer, inflammatory disorders, and metabolic diseases, leading to DNA, proteins, and carbohydrates damage, denoted as oxidative distress (Sies and Jones, 2020), Christian et al. (2014) have analyzed fifteen clinical failed BHV using mass spectrometry and found that levels of ortho-tyrosine, meta-tyrosine, and dityrosine conspicuously increase among failed BHV. Furthermore, 3-Chlorotyrosine, an oxidized amino acid formed by myeloperoxidase-catalyzed chlorinating oxidants, was correlated with BHV calcification (Lee et al., 2017). GLUT treated bovine pericardium modified with the antioxidant, 3-(4-hydroxy-3,5-di-tert-butylphenyl) propyl amine (DBP), showed significant reducing degree of calcification after implanted in the subdermal area of a rat model (Christian et al., 2015). And after exposed to H_2_O_2_ and FeSO_4_ to mimick the action of oxidative distress, GLUT treated bovine pericardium was detected with loss of GLUT crosslinking and morphology changes (Christian et al., 2015). Collectively, oxidative stress, causes collagen breakdown and a uniform susceptibility to collagenase for valve tissue, particularly via hydroxyl radical and tyrosyl radical mediated pathways, bring about BHV calcification.

#### 5.2.3 Metabolic Disorders

Several studies have indicated that BHV calcification is an atherosclerotic-like process. Namely, several factors involved in the pathogenesis of atherosclerosis and calcific aortic valve disease were also implicated in BHV calcification, especially lipid-driven factors (Wang et al., 2021a). Clinical researchers have stated that after BHV implanted, plasma levels of total-cholesterol, low-density lipoprotein-cholesterol (LDL), apolipoprotein B (ApoB), oxidized low-density lipoprotein (ox-LDL) were notably higher among patients with SVD than those without SVD (Mahjoub et al., 2013; Nsaibia et al., 2016; Nsaibia et al., 2018), and ApoB/ApoA-I ratio (Mahjoub et al., 2013) and OxLDL/HDL ratio (Nsaibia et al., 2016) could be considered as strong independent predictors of BHV failure. Taken together these findings all emphasize the pivotal role of lipid-mediated mechanism for BHV failure and calcification. Immunohistochemistry staining of failed BHV showed that ox-LDL was present in the fibrosa layer of BHV and surrounded by CD68-positive macrophages (Shetty et al., 2009). The crux of the lipid-mediated BHV degrading and calcification lies in the *in situ* formation of ox-LDL and subsequent activation of macrophages. After BHV implantation, high levels of circulating LDL deposit in the valve tissue where LDL is oxidized to ox-LDL. Ox-LDL is being phagocytosed by macrophages resulting in polarization and foam cell formation, and further retained by GAGs that infiltrated macrophages generate (Plakkal Ayyappan et al., 2016).

Despite lacking an in-depth understanding, some studies attempt to explain the lipid-mediated inflammatory mechanisms involved in BHV calcification. Shetty and colleagues deemed ox-LDL were bound and internalized by macrophages through CD36 (Shetty et al., 2009), a scavenger receptor with the highest affinity for ox-LDL (Miller et al., 2011). Activated lipid-laden macrophages form pseudopods, as well as produce cytokines and MMP9, finally resulting in weakening and calcification of the leaflet matrix. Additionally, elevated activity of lipoprotein-associated phospholipase A2 (Lp-PLA2) both in plasma and failed BHV tissues give support to the hypothesis that Lp-PLA2 takes part in BHV calcification (Mahmut et al., 2014; Mahmut et al., 2016). The primary source of Lp-PLA2 might be tissue macrophages instead of circulating leucocytes (Ferguson et al., 2012). Ox-LDL can upregulate Lp-PLA2 expression in monocytes/macrophages through the PI3K and p38 MAPK pathway (Wang et al., 2010). Lp-PLA2 rapidly cleaves oxidized phosphatidylcholine molecules produced during the oxidation of LDL, generating the soluble proinflammatory and lyso-phosphatidylcholine (Wilensky and Macphee, 2009), while the latter is a strong candidate for osteogenic stimuli (Vickers et al., 2010). Another function of ox-LDL is to induce the expression of PCSK9 in macrophages and stimulate Toll-like receptors (TLRs) (Nsaibia et al., 2018), promoting an osteogenic inflammatory response by activating the NF-κB pathway (Tang et al., 2012; Wang et al., 2021b). Collectively, ox-LDL is a substantial contributor to BHV calcification.

In addition to lipid disorder, metabolic syndrome (MS), also known as syndrome X, insulin resistance, etc, is an aggregate of clinical conditions characterized by central and abdominal obesity, systemic hypertension, and insulin resistance (McCracken et al., 2018). Previous research suggested MS is a strong independent predictor of bioprosthetic valve degeneration (Briand et al., 2006). And patients with type 2 diabetes mellitus undergoing bioprosthetic valve implantation are more susceptible to BHV calcification (Lorusso et al., 2012). Currently, the metabolic mechanisms responsible for BHV calcification are poorly defined, but oxidative stress secondary to diabetes mellitus is hypothetically involved in BHV calcification (Cote et al., 2010).

#### 5.2.4 Platelets and Subclinical Thrombosis

Bioprosthetic valve thrombosis is a rare but life-threatening complication that causes prosthetic valve obstruction (Brown et al., 2012). However, subclinical leaflet thrombosis occurred frequently, as it was detected in 12% of patients after BHV implanted (Chakravarty et al., 2017). Recently, literature illustrates that subclinical leaflet thrombosis is associated with BHV calcification. Cartlidge et al. (2019) observed a close spatial interaction of calcification with leaflet thrombosis and suggested thrombosis may be a potential upstream trigger for calcification.

Von Willebrand Factor (vWF) is the primary mediator of thrombosis, interacting with platelets. vWF is deactivated and cleaved by thrombospondin type-1 motif family and then maintained at a low concentration in the blood under high wall shear stress conditions in patients with aortic stenosis (Van Belle et al., 2019). Once the shear stress level was corrected after surgery, the concentration of vWF increased instantaneously (Sedaghat et al., 2017). The acute release of vWF promotes thrombus formation *in vivo*. Physiologically, the interaction of plasma VWF with platelets is induced by subendothelial collagen. In fact, the confluent endothelial layer is entirely lost in commercial BHV, so collagen type I, which is the main ECM component, is directly exposed to activate vWF. The formation of subclinical leaflet thrombosis induces inflammatory responses, valve fibrosis, and calcification. Moreover, a steep increase in calcium levels was shown on platelets when they were in contact with type 1 Collagen, possibly by activating calcium channels via phospholipase C and inositol 1,4,5 trisphosphate (Asselin et al., 1997; Roberts et al., 2004). The analysis above may help explain the relationship between thrombosis and calcification.

## 6 Potential Drug Therapy

Although BHV has been the mainstay of prosthetic valve substitutes for valve replacement surgery, mechanisms and pathogenetic factors of BHV calcification still being far from a clear elucidation of their nature, impeding the development of drug intervention to prevent or slow the process of BHV calcification.

No clinical drug targeting BHV calcification is currently available, however, statin treatment, both rosuvastatin, and atorvastatin could significantly diminish BHV calcification (Lorusso et al., 2010; Lee et al., 2019). In a rat subdermal implantation model, Sak Lee et al. (Lee et al., 2019) suggested that rosuvastatin attenuated BHV calcification associated though interleukin-6 and bone morphogenetic protein two downturns. Similarly, atorvastatin changes the global extent of inflammatory infiltrates but the proportions of the single inflammatory subsets, contributing to reduction of BHV calcification, either in terms of microcalcification or global calcium content (Lorusso et al., 2010). Some researchers showed that statin treatment is associated with significantly less BHV calcification and improved long-term outcomes (Antonini-Canterin et al., 2003; Sasaki et al., 2021). Yet, in one observational study by Kulik and colleagues, early lipid-lowering therapy did not lower BHV calcification (Kulik et al., 2010). Overall, statin treatment may be an effective therapeutic means after BHV implantation but requires more high-level evidence to support.

According to the linkages between immune response and BHV calcification, immunosuppressive therapy represented a potential candidate to delay BHV calcification and failure, especially for young patients. A study reported that the calcific degree of BHV in patients who had been given steroid treatment for aortitis was decreased (Eishi et al., 1996). Rabbit anti-thymocyte globulin (ATG) is a polyclonal IgG preparation used for induction treatment of immunosuppression used in malignancies, graft-versus-host disease, and autoimmune diseases, to decrease early rejection (Mohty, 2007). ATG treatment may induce long-lasting anti-Neu5Gc IgG responses with immune memory (Couvrat-Desvergnes et al., 2015; Amon et al., 2017). These researches reveal a potential therapeutic strategy for preventing BHV calcification, and ATG treatment immediately following surgery deserves more molecular mechanism research and clinically relevant trial in the future. However, due to the many side effects of immunosuppressive therapy, it should be carefully considered.

Currently, Oral anticoagulation for the first 3 months after surgical implantation of BHV is recommended according to the European Society of Cardiology/the European Association for Cardio-Thoracic Surgery and American College of Cardiology/American Heart Association Guidelines (Otto et al., 2021b; Vahanian et al., 2021). Since subclinical leaflet thrombosis may be a cause of BHV calcification, another possible treatment option could be considered is prolonged, even life-long use of anticoagulant drugs. But in such a way, the uppermost advantage of BHV over MHV would be weakened. So how long it is justified for anticoagulant treatment after BHV implantation merits further discussion.

Given the well-recognized association between end-stage renal disease, diabetes mellitus, hyperparathyroidism and BHV calcification, aggressive treatment should be applied. Notably, sevelamer hydrochloride, a phosphate binder used to treat hyperphosphatemia in patients with chronic kidney disease, has been showing the ability to decrease BHV calcification (Meng et al., 2021).

## 7 Conclusion and Outlook

In the past few decades, BHV possess significant advantages by alleviating the need for anticoagulation treatment and their exquisite hemodynamic properties after constant updating and optimization. However, the limited durability mainly due to SVD remains a challenging barrier to widen the scope of usage. Calcification is the most frequent presentation and the core pathophysiological process of SVD. Uncovering the basic mechanisms of BHV calcification is an essential prerequisite to address issues that currently exist.

**FIGURE 3:**
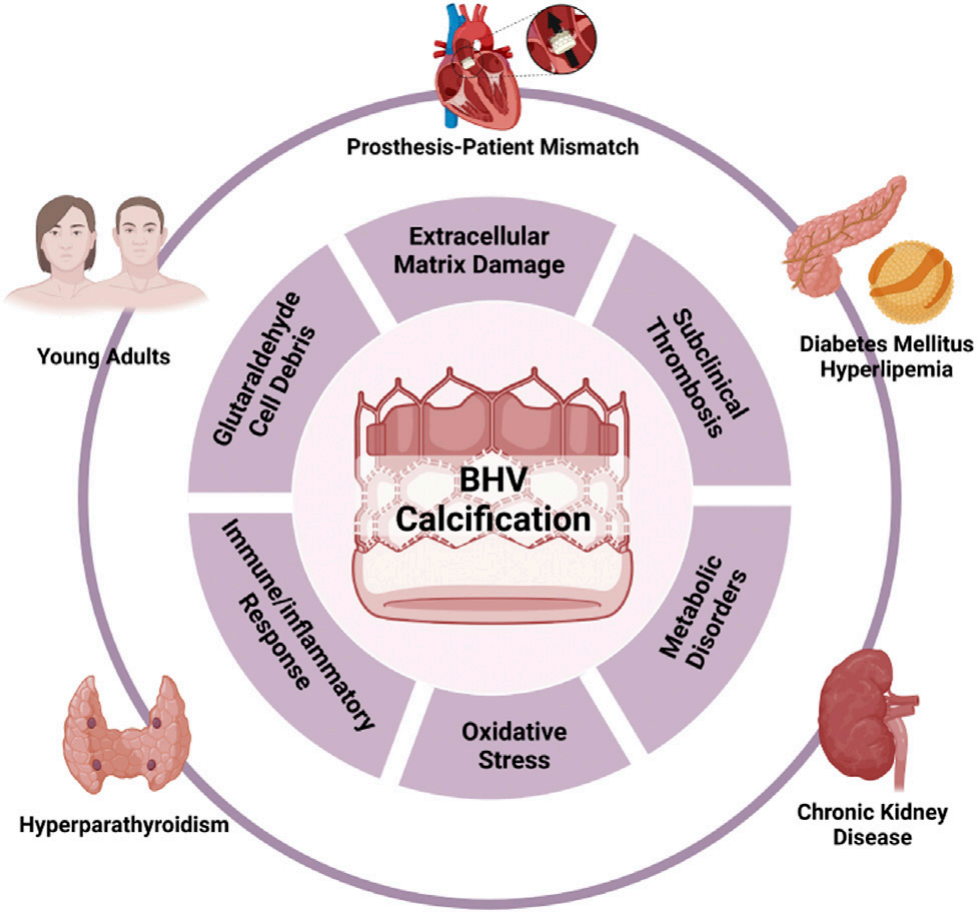
Risk factors and mechanisms of BHV calcification.

Mechanisms of BHV calcification are described in detail in the current review (Figure 3). In summary, residual cell debris after GLUT treatment and degeneration of ECM components are absolute necessities for BHV calcification. We also highlight the value of inflammatory reaction and immune response, oxidative stress, formation of ox-LDL, and subclinical leaflet thrombosis in the pathogenesis of BHV calcification. As alluded to above, just a few studies focusing on the pharmacological strategies of BHV calcification have been conducted, either animal or clinical studies. Currently, the potential therapies include lipid-lowering therapy, immunosuppressive therapy and aggressive treatment for comorbidities.

Basic research and explorations to obtain a better understanding of BHV calcification are still in their infancy. There are still a number of unknowns which require further exploration and discussion. First of all, the mechanisms leading to increased immune cells infiltration, and how this effect correlates with BHV calcification are unclear. As such, this should be a focus of subsequent research. Secondly, current known main cell types of pathological process in calcific aortic valve disease have been described in detail (Xu et al., 2020), but whether other cells than immune cells involved in BHV calcification still remains a certain. To shed more light on the mechanisms of BHV calcification, further studies will be dedicated to the unraveling of cell-type atlas and intercellular interactions. Thirdly, Screening for more effective drugs to prevent or delay BHV calcification warrants further research. In conclusion, the increasing demand for BHV implantation mandates enhanced the investment in BHV calcification research and the transition from bench to bedside.
